# Suicide Prevention Public Service Announcements Impact Help-Seeking Attitudes: The Message Makes a Difference

**DOI:** 10.3389/fpsyt.2016.00124

**Published:** 2016-07-15

**Authors:** Bonnie Klimes-Dougan, Nathan Wright, David A. Klingbeil

**Affiliations:** ^1^Department of Psychology, University of Minnesota, Minneapolis, MN, USA; ^2^State of Minnesota: Department of Public Health, Minneapolis, MN, USA; ^3^Department of Educational Psychology, University of Wisconsin-Milwaukee, Milwaukee, WI, USA

**Keywords:** suicide prevention, public service announcements, billboards, universal prevention, young adults, help-seeking

## Abstract

Suicide continues to be one of the most serious public health challenges. Public service announcements (PSAs) are frequently used to address this challenge, but are rarely sufficiently evaluated to determine if they meet the intended goals, or are associated with potential iatrogenic effects. Although it is challenging to assess the relative impact of different PSA modalities, our group previously noted that one billboard message failed to show the same benefits as one TV ad [e.g., Klimes-Dougan and Lee (1)]. The purpose of this study was to extend these findings to test critical aspects of suicide prevention billboard messaging. Although both simulated billboard messages presented had identical supporting messages, we predicted that the more personal billboard message, focused on saving one’s life, would cause more favorable help-seeking attitudes than the message focused on suicide. Young adult university students (*N* = 785) were randomly assigned to one of three conditions; one of two billboard simulations or a TV ad simulation. Help-seeking attitudes, maladaptive coping, and reports of concern and distress were evaluated. The results of this study suggest some relative benefits in endorsement of favorable help-seeking attitudes for one of the billboard conditions – stop depression from taking another life. Although further research is needed to determine what methods will alter the risk for suicide in the population, the results of this study provide a useful first step showing that some billboard messaging may favorably influence help-seeking attitudes.

## Introduction

Suicide is a significant contributor to lost years of life among young adults, and it was the second leading cause of death among 15–29 years olds in 2012 (2). The importance of implementing suicide prevention programs is understood and widely accepted, but preventing suicide remains an elusive goal. It is likely that suicide prevention efforts will benefit from the inclusion of a range of efforts from relapse prevention, means restriction, to other universal preventive interventions (3). The focus of this paper is on universal prevention programs, those programs that are dispersed across a population. Specifically, the focus is on public awareness campaigns. Despite the proliferation of suicide prevention campaigns over the last three decades, many of the campaigns have not been rigorously studied and lack an empirical basis (4, 5). Some suicide prevention efforts fail to produce the desired outcomes. Some approaches may even inadvertently produce iatrogenic effects. Possible iatrogenic effects include messages that normalize suicidal behavior, reinforce biases that prevent individuals from seeking help, or heighten susceptibility for suicide contagion [e.g., Ref. (5, 6)]. More research is needed to validate the effectiveness of suicide prevention campaigns, before taking them to scale.

There are numerous challenges to evaluating suicide prevention programs. For example, it is difficult to show treatment effectiveness among infrequently occurring behaviors, such as deaths by suicide or even suicide attempts (7). The U.S. Surgeon General has called for researchers to develop alternative methods for evaluating suicide prevention efforts beyond simply a reduction in the number of suicides (8). Intermediate measures of intervention effectiveness include assessing changes in help-seeking attitudes and behaviors (9, 10).

Help-seeking is defined as the act of seeking assistance from an informal (e.g., friend) or formal (e.g., mental health professional) source (11). Help-seeking is thought to be a critical step between understanding that there is a problem and seeking out the necessary services for assistance (12). Barriers to help-seeking include stigma and other maladaptive beliefs [e.g., Ref. (13, 14)]. Increased help-seeking is associated with lower suicide risk (15). If prevention programs are to succeed, help-seeking must be promoted. And yet, there is mixed evidence if suicide prevention efforts are effective at increasing help-seeking attitudes and behaviors, especially in those who are at high risk for suicide (9). Similarly, despite the evidence that many individuals who experience depressive symptoms or suicidal ideation use more maladaptive coping and isolative behaviors (15), there is mixed evidence if preventive interventions alter maladaptive coping strategies (16, 17). It is a priority to promote enhanced adaptive attitudes and behaviors.

There is a limited understanding of what features of suicide prevention public awareness campaigns show the most impact. Some strides have been made in assessing the acceptability and impact of public service announcements (PSAs) through the media (e.g., TV ads, radio ads, websites) or printed material (e.g., billboards, flyers, posters). Often these campaigns target white, adult males, the group that accounts for the highest risk of suicide, or target significant others who may serve as sources of referral (e.g., wives, girlfriends, friends). For example, Daigle et al. (18) measured the efficacy of a multimedia-based prevention approach (*via* radio, television, and billboards) intended at reducing suicide rates among males in Quebec, Canada. They exclusively evaluated the results of this campaign and targeted a male audience (e.g., *Pain is not gender-specific – yet 80% of suicides are committed by men*). Male participants exposed to the campaigns gained more knowledge of suicide facts and resources, yet there was no evidence that the campaigns impacted the participant’s intention to utilize mental health services.

Given that universal prevention efforts are viewed by all ages, the impact has also been assessed in other groups, even for groups that were not the target of the campaign. That is, while the target group is rarely adolescents or young adults [for an exception, see Ref. (19)], research is needed for this age group as they are at highest risk of iatrogenic effects of suicide prevention efforts [e.g., Ref. (6)]. A series of simulation studies evaluated the benefits and risks associated with viewing a PSA. In one study, high school students were randomly assigned to a billboard, a TV ad, or a no information condition. The results revealed some concerns about the effectiveness of PSAs, particularly the billboard condition with high-risk youth (20). When this study was conducted with college students, those in the billboard condition were less likely to endorse adaptive help-seeking attitudes than those in the no information condition or in the TV ad condition (1). The results of these findings led the authors to conclude that this particular billboard was largely without benefits. The researchers also noted that it remains unclear if the modality (billboard) or message (e.g., content of the message) accounted for the unfavorable outcomes associated with viewing this billboard.

The purpose of this study was to examine individual differences in help-seeking attitudes, knowledge of maladaptive coping behaviors, and reported concerns about PSA exposure among young adults in response to two different simulated billboard messages and a simulated TV ad. Here, our goal was to evaluate if subtle features of the simulated billboard message made a difference. The two messages assessed were identical in every way (e.g., the call to action was *see your doctor*) with the exception of the central message (either the original message – *Prevent Suicide: Treat Depression* or the alternative message – *Stop depression from taking another life*). The wording on the alternative billboard was intended to motivate the viewer by being more personal, by stressing the benefits valued by the intended audience that offset the costs of taking action (21), and by having the viewer consider the implicit directive of acting to save one’s life. Additionally, the alternative billboard was intended to decrease psychiatric jargon and avoid the possible stigma associated with the word “suicide” (22). The hope is that this work will promote a better understanding of the utility of campaign messages so that the intended goal of saving lives can be more fully realized.

## Materials and Methods

### Participants

A total of 785 part- or full-time university students between the ages of 18 and 34 years old (M = 21.9; SD = 2.8) served as participants for this study. The sample consisted of primarily upper level undergraduate students (81.6%). The majority of participants were females (79.2%). The majority of participants primarily identified as Caucasian (65.2%), followed by Latin American (21.9%), Native American (6.3%), Asian American (4.5%), and African American (1.4%). Nearly 89% of participants were born in the United States. There were 406 participants in the original billboard group, 279 participants in the alternative billboard group, and 100 participants in the TV ad group. Because the focus of this study is on young adult perceptions, those who were 35 years of age or older were excluded from this study (*N* = 30).

### Dependent Variables

#### Help-Seeking Attitudes and Maladaptive Coping

The help-seeking attitudes and endorsement of maladaptive coping behavior scales were adapted from Gould et al. (15), who reported Cronbach’s alpha coefficients of 0.60 for the help-seeking and 0.54 for the maladaptive coping scale based on scales derived from factor analysis.

The five-item help-seeking attitudes scale required participants to rate their response to a suicidal friend on a five-point scale (0 = “never” to 4 = “always”) on a number of help-seeking behaviors (i.e., get advice from another friend, tell my friend to see a mental health professional, tell my friend to call a hotline, talk to an adult about my friend, tell my friend to talk to his or her parents). An average help-seeking score was calculated for each participant. The score used in the analysis, which ranged from 0 to 4, reflects the participant’s average endorsement of help-seeking attitudes.

Participants were asked to indicate if they agreed or disagreed with seven statements that reflected maladaptive coping strategies (i.e., people should be able to handle their own problems without outside help, suicide is a possible solution to problems, if you are depressed it is a good idea to keep your feelings to yourself, drugs and alcohol are a good way to help someone stop feeling depressed, keep it a secret, talk to my friend without getting anyone else’s help, I would not take it seriously). Three items were on the same five-point scale as the help-seeking questions that were converted to agree-disagree statements. The total number of maladaptive coping behaviors endorsed was calculated for each participant.

#### Concern or Distress

An additional item was used to evaluate if viewing the PSAs resulted in feelings of concern and/or distress. Participants rated their level of concern using a five-point scale (“none” to “a lot”).

### Covariates

#### Demographic/Screening Questionnaire

The Demographic and Screening Questionnaire included questions regarding sex (male = 0; female = 1), age, and race/ethnicity, based on endorsement of one of six groups (European American-Caucasian, African American, Asian American, Latin American/Hispanic, Native American). This was later reduced (non-Caucasian = 0, Caucasian = 1). To assess the experience with depressive symptoms, participants were asked – “Have you felt sad almost all of the time or been depressed?” and if yes, “Have you gone to see someone (your doctor, a counselor, etc.) when you were depressed?” To assess suicide attempts, participants were asked – “Have you ever done anything to try to kill yourself (attempted suicide)?” These items were adapted from the wording of structured diagnostic interviews and a screening tool developed by Kroenke et al. (23). A broader index of risk status was based on those who screened positive for experience with depressive symptoms, and/or suicide attempts were considered high risk (low-risk status = 0 vs. high-risk status = 1). Additionally, a more narrow index of risk status, based on suicide attempt history, was considered for some follow-up analyses (no suicide attempt = 0 vs. suicide attempt = 1).

### Simulated PSA Exposure

All types of messaging tested here were developed by Suicide Awareness Voices of Education (SAVE), a Minneapolis-based non-profit suicide prevention agency, as part of a statewide public service campaign. In both billboard conditions, participants were asked to imagine they viewed it while driving in a vehicle. They were then shown a large PowerPoint projection (approximately 3 × 5 ft) of the billboard for 5 s. The original billboard read “*Prevent suicide. Treat depression*.” The alternative billboard read “*Stop depression from taking another life*.” Both billboards provided the directive, “*See your doctor*” and had the SAVE website listed. They also had both, the same depiction of a middle-aged, white male on the right of the billboard and a cardiac rhythm depicted along the bottom border.

Similarly, in the TV ad condition, participants were asked to imagine they saw the PSA while watching television. They were then presented with a 30-s video that featured several adults of different sexes and races. The video described depression as “*a brain illness*,” and noted salient symptoms of depression. The message went on to have components of both billboards (“*If you see the symptoms of depression, get that person to a doctor. With medical help, depression can be treated, suicide can be prevented. Learn how to stop depression from taking another life*.”). The video ended with the printed message “*Prevent suicide. Treat depression*.” with the phone number of SAVE.

### Procedure

The Institutional Review Board at the University of Minnesota approved this research proposal. Recruitment took place between 2006 and 2011. Participants were recruited from 17 behavioral science courses at the University of Minnesota. They were told this study examined the impact that PSAs have on suicide and depressive symptom knowledge, perceptions, and behaviors. The experiment was carried out during or after class. Participation was voluntary, and alternative class assignments were available for those who chose not to participate in this study. All participants completed a brief Demographic/Screening Questionnaire. Participants were then randomly assigned to conditions. Students were either randomly assigned to one of the two billboard conditions or one of the three conditions (original billboard, TV ad, and no information). Participants were generally asked to wait outside the classroom when their condition was not being shown.

Immediately after participants were exposed to the simulated PSA (i.e., billboards or TV ad), they were asked to complete the Suicide Awareness Questionnaire. The questionnaire was adapted for this study to evaluate participants’ (1) perceptions of the utility of PSAs, (2) knowledge of depressive symptoms, (3) normative beliefs (i.e., estimates of suicidal risk), and (4) knowledge of help-seeking and maladaptive coping attitudes. The focus here is on attitudes, but for more detailed information on the components of the Suicide Awareness Questionnaire, see Ref. (1).

To ensure participant anonymity, consent forms were completed and filed separately from questionnaires. Upon completion of the study, the researchers provided information about the university mental health services in the event that participants experienced distress from participating in research on the topic of suicide. The results pertaining to the no information condition are reported in Klimes-Dougan et al. (1) and are not included here. Instead, the focus here was on clarifying differences across PSAs. Study participation rate was 92%.

### Data Analyses

We used SPSS v. 22 (24) to conduct all the analyses. First, we examined the descriptive statistics for the demographic variables (e.g., sex, age, risk status) for the entire sample and then for the three conditions (see Table 1). Second, we calculated product moment correlations, point-biserial correlations, or phi coefficients to estimate the relationship between the potential covariates (i.e., demographic variables) and the dependent variables. Third, we conducted a series of multivariate regression analyses to consider the relationship between PSA conditions and reported help-seeking attitudes, endorsement of maladaptive coping behaviors, and reports of concern/distress after viewing the PSA. We included covariates based on their correlations with the dependent variables (age, sex, and race) and those that differed across group (risk status). Finally, we conducted a series of exploratory follow-up analyses to see if there was a significant interaction between risk status (with both the broad index – low risk vs. high risk and also the narrow index – no suicide attempt vs. suicide attempt) and group membership. We included covariates that were significant predictors from the regression models in these follow-up analyses. For all the analyses, the criterion for statistical significance was set at *p* < 0.05.

**Table 1:** **Participant demographics by condition**.

Characteristic	Original billboard	Alternative billboard	Video	*F*/χ^2^	*P*
*N*	406	279	100		–
Sex (female)	77.8%	79.2%	85.0%	2.51	0.286
Mean age (SD)	21.8 (2.7)	21.7 (2.7)	22.6 (3.3)	4.39	0.013
Race (Caucasian)	75.8%	75.5%	83.0%	2.61	0.272
Depression/suicide risk^a^	36.2%	27.2%	31.0%	6.17	0.046
Previous suicide attempt^b^	7.4%	6.8%	7.0%	0.08	0.961

*^a^Participants who indicated previous experience with depression and/or suicide were identified as high risk*.

*^b^Participants who indicated a previous suicide attempt were identified as high risk*.

## Results

### Descriptive Statistics and Preliminary Analysis

Demographic information for participants in each condition is presented in Table 1. The PSA groups did not differ on the percentage of female participants, the percentage of Caucasian participants, or on the number of reported previous suicide attempts. However, there were significant differences for participant age and the percentage of high-risk participants with previous depression or suicide attempt (although not for the narrowly defined suicide attempts).

Among all participants, the average score on the help-seeking scale was 2.72 (SD = 0.64), suggesting that they were likely to “sometimes” endorse the use of help-seeking behaviors for a friend. Skewness (−0.28) and kurtosis (0.24) statistics, along with visual inspection of a histogram and quartile plots, indicated that participant responses reasonably approximated a normal distribution.

By contrast, responses on the maladaptive coping strategies scale and the concern/distress question were skewed. Participants indicated if they agreed or disagreed with seven statements that represented maladaptive coping strategies. The range of responses was from 0 to 4. Approximately 48% of participants did not endorse a maladaptive coping strategy, 32% of participants endorsed one strategy, 14% endorsed two strategies, 5% endorsed three strategies, and 1% endorsed four strategies. The median response was endorsing one of the seven maladaptive coping strategies. Participants’ responses were positively skewed (1.07) and slightly leptokurtic (0.57). For the following analyses, we recoded the maladaptive coping score as a dichotomous variable (0 = no endorsement of maladaptive strategies; 1 = endorsement of one or more maladaptive coping strategies).

Participants were also asked to rate their level of concern/distress after viewing the PSA on a 1 to 5 scale. The median response was 1 (“none”) (M = 1.6, SD = 0.68), indicating a low level of concern or distress after viewing the PSA across all participants. Responses were positively skewed (1.92) and leptokurtic (4.11). Due to the skew of the data, the concern/distress score was recoded as a dichotomous variable (0 = no concern/distress or 1 = at least some concern/distress).

### Correlations

As shown in Table 2, correlations were generally weak across all variables. In this sample, the males were slightly older; there were more females who endorsed a history of depression or suicidal behavior and more non-Caucasians with a history of suicide attempt. Sex, age, and race were significantly associated with the average help-seeking score. Females, younger adults, and Caucasians were more likely to endorse help-seeking attitudes. Sex, race, and reported history of suicide attempts were significantly associated with the dichotomized maladaptive coping score. Males, non-Caucasians, and those with a history of suicide attempts were more likely to endorse maladaptive coping attitudes. Sex and race were associated with concern/distress after viewing the PSA. Females and Caucasians were more likely to endorse concern/distress. There was a negative relationship between participants’ average help-seeking score and dichotomized maladaptive coping score. Concern/distress after viewing the PSA was not associated with the help-seeking attitudes or maladaptive coping strategies.

**Table 2:** **Correlations matrix of dependent variables and covariates**.

	Sex^a^	Age	Race^b^	High-risk depression/suicide^c^	High-risk suicide attempt^d^	Average help-seeking scale	Dichotomized maladaptive coping score	Dichotomized concern or distress
Sex (male vs. female)	–							
Age	−0.082*	–						
Race (Non-Caucasian vs. Caucasian)	−0.021	−0.047	–					
High-risk depression/suicide	0.092*	0.069	0.028	–				
High-risk suicide attempt	0.081*	0.023	−0.082*	0.402*	–			
Average help-seeking score	0.177*	−0.080*	0.096*	−0.052	0.010	–		
Dichotomized maladaptive coping score	−0.168*	0.040	−0.147*	0.033	0.086*	−0.233*	–	
Dichotomized concern or distress	0.099*	−0.057	−0.095*	−0.043	−0.017	0.001	−0.001	–

*Reported correlations are product moment correlations (between two continuous variables), point-biserial correlations (between a continuous and dichotomous variable), or Phi coefficients (between two dichotomous variables)*.

*^a^Male = 0, Female = 1*.

*^b^Non-Caucasian = 0, Caucasian = 1*.

*^c^A broader index of risk status was based on those who screened positive for experience with depressive symptoms and/or suicide attempts were considered high-risk*.

*^d^A more narrow index of risk status was based on suicide attempt history was considered for some follow-up analyses*.

^*^p < .05.

### Help-Seeking Attitudes

For the PSA groups, average help-seeking attitudes were 2.64 (SD = 0.64) for the original billboard group, 2.81 (SD = 0.59) for the alternative billboard group, and 2.83 (SD = 0.68) for the video group. To examine the effect of PSA group, we fit a linear regression model that controlled for participants’ sex, age, race, and broad risk status. The overall model was statistically significant, *F*(6, 776) = 9.237, *p* < 0.001 (see Table 3). The covariates accounted for approximately 6% of the variance in participants’ help-seeking attitudes. Sex and race were the significant predictors of average help-seeking scores. The effect of age or depression/suicide risk was not significant after controlling for the effect of the other covariates in the model. After controlling for the covariates, there were significant differences between the PSA groups. Participants in the alternative billboard group and the TV condition endorsed significantly higher help-seeking attitudes than participants in the original billboard group.

**Table 3:** **Predictors of help-seeking attitudes**.

	*B* (SE)	95% CI	*t*	*p*
Constant	2.645 (0.197)	2.258, 3.033	13.408	<0.001
Group				
Video vs. original billboard	0.167 (0.069)	0.030, 0.303	2.401	0.017
Alternative billboard vs. original billboard	0.152 (0.048)	0.057, 0.247	3.154	0.002
Female (vs. male)	0.283 (0.055)	0.175, 0.391	5.149	<0.001
Age	−0.014 (0.008)	−0.030, 0.002	−1.712	0.087
Caucasian (vs. non-Caucasian)	0.144 (0.052)	0.042, 0.247	2.760	0.006
High-risk depression/suicide	−0.076 (0.048)	−0.170, 0.170	−1.605	0.109

*The overall model was significant, *F* = 10.55, *p* < 0.001. The predictors in the model accounted for 5.9% of the variance (adjusted *r*^2^ = 0.059) in average help-seeking scores*.

### Maladaptive Coping Strategies

Among the PSA groups, approximately 54% of participants in the original billboard group, 53% in the alternative billboard condition, and 42% in the TV ad condition endorsed one or more maladaptive coping strategies. To examine the effect of PSA group, we fit a logistic regression model (using the logit-link function) that included age, sex, race, and risk status as covariates. For the categorical variables, the original billboard group, females, Caucasians, and participants with low risk were coded as the referent groups.

Results from the Hosmer–Lemeshow test suggested that the model adequately fit the data (χ^2^ = 8.486, *p* = 0.387). Including the covariates improved the classification accuracy from the unconditional model by 8.5%. Male participants, OR = 2.46, 95% CI (1.69, 3.58) and non-Caucasian participants, OR = 2.05, 95% CI (1.44, 2.92) had significantly increased odds (*p* < 0.001) of endorsing one or more maladaptive strategies after controlling for other variables in the model. Participants endorsing previous suicide risk were also more likely to endorse one or more maladaptive coping strategies, OR = 2.12, 95% CI (1.17, 3.84), *p* = 0.013. Age was not a significant predictor. After controlling for these covariates in the model, there was no significant difference in the odds of endorsing one or more maladaptive strategies when comparing the original billboard to the TV ad condition (OR = 0.67, *p* = 0.08) or the original billboard to the alternative billboard (OR = 1.00, *p* = 0.98) condition.

### Concern/Distress

Some level of concern or distress after viewing the PSAs was endorsed by about a third of the sample. Thirty-one percent of the participants in the original billboard group, 33% in the alternative billboard group, and 29% in the TV ad group reported some concern/distress after viewing the PSAs. We fit a logistic regression model (using the logit-link function) to examine the effect of PSAs after controlling for sex, race, and risk status. Results from the Hosmer–Lemeshow test suggested that the model adequately fit the data (χ^2^ = 10.59, *p* = 0.16), but including the covariates did not improve the classification accuracy from the unconditional model. Females, OR = 1.79, 95% CI (1.19, 2.71), *p* = 0.005, and Caucasians were significantly more likely to report concern/distress, OR = 1.57, 95% CI (1.11, 2.23), *p* = 0.011, after controlling for the other variables in the model. Depression/suicide risk did not have a significant effect on the probability of indicating concern or distress, OR = 0.79, *p* = 0.17. After controlling for the covariates, viewing the TV ad condition compared to the original billboard (OR = 0.92, *p* = 0.75) or the alternative billboard compared to the original billboard (OR = 1.08, *p* = 0.66) did not have a significant effect on the odds of reporting concern/distress.

### Exploratory Analyses

There were no significant interactions for risk status (either using the broad or narrow index) and group status for any of the outcome variables.

## Discussion

Universal suicide prevention efforts hold great promise in reaching many in need. Nevertheless, the lessons learned in research conducted over the last 30 years have curtailed enthusiasm for PSAs, as these campaigns have limitations for communicating critical information and potential iatrogenic risks [e.g., Ref. (5, 25)]. Emerging evidence suggests that theory-driven development and empirical assessment of message content for suicide should be the gold standard (26), but the current state of the field is inchoate (27). Additionally, because PSA exposure may not be limited to the target population, it is also critical to determine the efficacy and safety for other potentially vulnerable populations who may incidentally view PSAs. It is possible that specific formats or messages may have an important impact on the viewer. While progress in developing effective suicide prevention PSAs may be uneven, it is likely that using theoretically guided, practical steps to validate the effectiveness of PSAs will ultimately prove to be beneficial for the society.

The results of this study replicate and extend previous research regarding the utility of PSAs. Specifically, the findings are consistent with previous work showing that the original billboard was not as effective as the TV ad [e.g., Ref. (1, 20)]. The TV ad conveys information about the common symptoms of depression, imbeds the topic of suicide into a broader discussion of depression, and provides more extensive information about services for treating depressive symptoms and preventing suicide. Increasing the individuals’ knowledge and awareness of services has been found to be a crucial factor in ensuring media messages are acted on (28).

Importantly, the results of this study suggest that some billboard messages may yield changes in attitudes that are nearly comparable to the TV ad. Specifically, in this study, we showed that attitudes toward help-seeking were favorably influenced by the alternative billboard message (“Stop depression from taking another life”), but not the original message (“Prevent suicide: Treat depression”). Previously, Klimes-Dougan and Lee (1) speculated that some billboards may contain an insufficient “dose” of information for changing attitudes. But the results of this study suggest that the amount of information conveyed in a billboard may not be a critical factor. In this study, all wording was exactly the same in both the billboards other than the central message (so, in total, there were only two words more in the alternative billboard). By contrast, the findings of this study suggest that brief messaging may be useful if the wording of the message is crafted to maximize benefits for the viewer. Some have pointed out the advantages of using a straightforward and succinct message [e.g., Ref. (29)]. It will be important to better understand the features of the alternative billboard message – “*Stop depression from taking another life*” that are most useful. Not only is this message succinct but also it may be more personally relevant, more appealing, and possibly even, inspire intervention. Additionally, the less explicit reference to suicide in the alternative billboard message may be less off-putting. Nevertheless, it remains somewhat surprising that in some respects, the alternative billboard shows similarly favorable outcomes as the TA ad, especially given the depth of information that is conveyed in the TV ad.

Contrary to the predictions, we failed to show that at-risk participants were more vulnerable to the negative effects of any of the simulated PSAs. Previous research with PSAs has shown that those who reported depressive symptoms and/or previous suicide attempts were the least likely to endorse positive help-seeking attitudes and the most likely to see a strong link between depression and suicide, particularly for the original billboard (20). The results of this study fail to show evidence that the responses differed across PSA conditions. However, consistent with other work that shows an association between depressive symptoms and help-seeking attitudes or behaviors (15, 30, 31), the results of this study did suggest that the most vulnerable individuals were more likely to endorse maladaptive coping strategies. Future research should continue to examine the utility of personalization for suicide prevention campaigns, for what might be useful for those at high risk may not be useful for those at low risk for suicide.

There are some important contributions of this study as well as interpretational cautions that should be highlighted. The randomized assignment of the participants to exposure conditions (despite some variations in randomization procedures across classrooms) provides an important design that lends itself to causal implications. Research using a pretest and posttest design may further aid conclusions by showing how outcome variables change after viewing a PSA. Additionally, the relatively large, homogeneous group of young adult participants provided sufficient power to detect small effect sizes. There are measurement issues that need to be addressed in future research, given the moderate internal consistency of the scales (e.g., maladaptive coping), the distribution of responses, and the inconsistent findings across scales (despite the fact that the maladaptive coping scale is related to the help-seeking scale, *r* = −0.23). Additionally, this study examined endorsed attitudes after viewing a PSA, and not the actual behaviors. It would have been ideal to also know if young adults who viewed PSAs were more likely to reach out to family members, friends, or professionals to get help for themselves or others who are struggling with suicidal thoughts. Second, there are limitations regarding generalizability of the results given that the majority of the participants were white, female college students selected from behavioral science courses. In some respects, the characteristics of these participants may have been ideal. Namely, this campaign was specifically intended for females (e.g., friends, spouses etc., of those who are at high risk for suicide – white males) so it may be useful to have a greater proportion of females, particularly at-risk females. However, given that, we know suicide prevention efforts differ for males and females (32, 33) and there are robust sex differences for suicide attempts and deaths, a more equally distributed sample of males and females might have been preferable in some respects.

It will be important to ask the question, how representative are PSA simulation studies of real-world conditions? While simulation designs provide well-controlled exposure (e.g., a 5 s viewing of the simulated billboard), the exposure is likely to vary significantly under natural conditions. For example, participants viewing a billboard from a car may be focused primarily on traffic, perhaps only briefly glancing at the message or missing the message completely. Increasingly, cluttered media environments make achieving adequate exposure to media campaigns more difficult, rather than making wider exposure easier (28). Also, in this study, there was only one exposure to this simulated billboard message. In real-world contexts, some people may experience repeated exposures to the messaging, potentially based on the placement of the billboards and an individual’s transportation patterns or TV viewing patterns. The salience of the messaging is also important. But, while some public health topics provide provocative messaging that is readily recalled (as the recent “Cover your Butt” colonoscopy billboard campaign), it is incumbent upon preventionists to be cautious in suicide prevention messaging. Finally, continued scrutiny of the messages may be worthwhile. For example, contrary to suicide messaging guidelines (34), there may be some ways in which the imperative of the alternative billboard is not ideal. For example, “ … *taking another life*” may imply that that lives are “taken” with some regularity and that suicide is normative. It will be important to continue to refine messaging strategies and back these modifications with empirical evidence. Given that messaging may not be uniformly effective across participants, future research should also address the important questions of which suicide prevention approaches are best, and which are best for whom. Due to the significant impact suicide continues to have on the lives around the world, further research is needed to develop and improve effective suicide prevention campaigns so that more can benefit.

## Conclusion

Suicide remains a serious public health challenge worldwide and is among the top causes of death for young adults (2). There is great promise in media campaigns as they afford the opportunity to present well-defined messages to large audiences repeatedly, over time, at a low cost. However, this promise is rarely realized because media campaigns can fall short or even backfire (28). In an effort to do something, preventionists have often moved ahead in the absence of firm empirical grounding. The results of this study provide some optimism that carefully crafted billboard messages may favorably influence help-seeking attitudes of participants.

## Author Contributions

BK-D was the PI on this project and oversaw all aspects of this study and was the primary contributor to the conceptualization and writing of this manuscript. NW conducted preliminary data analysis on a related topic using these data for his thesis and contributed to the preliminary conceptualization, data analysis, and writing of this early version of this manuscript. DK contributed to the conceptualization and data analysis of this manuscript.

## Conflict of Interest Statement

The authors declare that the research was conducted in the absence of any commercial or financial relationships that could be construed as a potential conflict of interest.
